# Association between Complete Proteinuria Remission and Kidney Function in the Phase 3 PROTECT Trial of Sparsentan in IgA Nephropathy

**DOI:** 10.2215/CJN.0000000961

**Published:** 2025-12-22

**Authors:** Hiddo J.L. Heerspink, Brad H. Rovin, Radko Komers, Bruce Hendry, Alex Mercer, Priscila Preciado, Edward Murphy, Vladimir Tesar

**Affiliations:** 1 Department of Clinical Pharmacy and Pharmacology, University of Groningen, University Medical Center, Groningen, The Netherlands; 2 University Wexner Medical Center, Columbus, Ohio; 3 Travere Therapeutics, Inc., San Diego, California; 4 JAMCO Pharma Consulting, Stockholm, Sweden; 5 General University Hospital, Charles University, Prague, Czech Republic

**Keywords:** angiotensin, ESKD, glomerular disease, GFR, IgA nephropathy, kidney failure, proteinuria, randomized controlled trials, renal function, renal protection

## Abstract

**Key Points:**

- This analysis explored the association between complete remission of proteinuria and kidney function decline during sparsentan or irbesartan treatment.
- Patients achieving complete remission of proteinuria experienced better eGFR preservation and fewer kidney failure events.
- These data support Kidney Disease Improving Global Outcomes guideline recommendations to maintain proteinuria levels in IgA nephropathy ideally below 0.3 g/d.

**Background:**

In the phase 3, randomized, double-blind PROTECT (NCT03762850) trial, sparsentan, a single-molecule dual endothelin angiotensin receptor antagonist, reduced proteinuria and preserved kidney function compared with maximum labeled dose irbesartan in adults with IgA nephropathy. In this *post hoc* analysis of PROTECT, we assessed the association between complete remission of proteinuria (CR) and preservation of kidney function.

**Methods:**

This analysis compared kidney function in patients who achieved CR (urine protein excretion <0.3 g/d) by week 36 (CR36) or at any time up to week 110 (CR110) versus those who did not (non-CR), regardless of original treatment allocation. End points assessed by CR status were change in proteinuria, eGFR, and BP, rate of eGFR decline, a composite kidney end point, and safety.

**Results:**

Of 404 patients who were randomized and received study drug, 43 (11%) achieved CR36 and 85 (21%) achieved CR110. CR patients demonstrated greater and more rapid reductions in proteinuria compared with non-CR patients. CR110 patients had a smaller absolute change in eGFR versus non-CR patients (−4.0 versus −8.6 ml/min per 1.73 m^2^) and a slower rate of eGFR decline (day 1–week 110; −0.7 versus −4.2 ml/min per 1.73 m^2^ per year). Fewer CR110 patients (1%) reached the composite kidney end point versus non-CR patients (14%). CR110 patients were more likely to experience treatment-emergent adverse events associated with hypotension (hypotension, orthostatic hypotension, or BP systolic decreased) and less likely to experience treatment-emergent adverse events of hypertension than non-CR patients. More non-CR patients versus CR110 patients discontinued treatment due to AEs (11% versus 4%, respectively) or patient decision (8% versus 2%, respectively).

**Conclusions:**

Participants in PROTECT who achieved CR36 or CR110 showed greater eGFR preservation, fewer kidney failure events, and similar safety profiles compared to non-CR participants. These data reinforce recommendations to maintain proteinuria levels ideally <0.3 g/d and underscore its relationship with kidney function preservation.

**Clinical Trial registry name and registration number:**

ClinicalTrials.gov, NCT03762850.

## Visual Abstract

**Figure:**
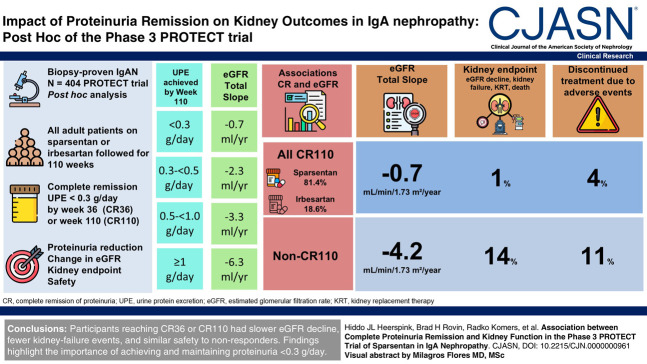

## Introduction

IgA nephropathy, a rare primary glomerular nephritis, has often been considered a relatively benign disease and can be asymptomatic.^1^ However, in some patients, IgA nephropathy can progress to kidney failure rapidly within years.^1^ Recent observational studies have shown that the presence of significant proteinuria, even at levels traditionally considered to be low risk, is associated with a high likelihood of kidney failure.^2^ In addition, reduction of proteinuria to low levels including <0.3 g/d has been associated with a lower risk of kidney failure in multiple cohort studies.^2–6^ Data from clinical trials also demonstrated an association between early treatment effects on proteinuria and treatment effects on eGFR decline and clinical kidney outcomes, supporting the use of proteinuria as a reasonably likely surrogate end point in clinical trials of IgA nephropathy.^7,8^

The phase 3 PROTECT trial enrolled 404 participants with IgA nephropathy at high risk of progression (defined as proteinuria at least 1.0 g/d) despite maximum labeled renin-angiotensin system (RAS) inhibition.^9,10^ The trial was designed to investigate the safety and efficacy of sparsentan, a single-molecule dual endothelin angiotensin receptor antagonist (DEARA) that targets both endothelin and angiotensin II receptors (ET_A_R and AT_1_R, respectively).^9^ Sparsentan significantly reduced proteinuria compared with maximum labeled dose irbesartan by 41% after 9 months of treatment. This beneficial effect was sustained through 2 years follow-up at which point eGFR decline was 3.8 ml/min per 1.73 m^2^ less compared with irbesartan treatment.

In the PROTECT trial, many patients achieved complete remission of proteinuria (CR; urine protein excretion [UPE] <0.3 g/d), and this was achieved more frequently with sparsentan compared with irbesartan. PROTECT thus provides an excellent platform to test the effect of achievement of proteinuria remission thresholds (yes versus no) on preservation of kidney function in patients with IgA nephropathy in the setting of a rigorous registrational clinical trial.^9^

In this *post hoc* analysis of the PROTECT trial double-blind period, we therefore assessed the effect of achieving versus not achieving CR on kidney function decline (assessed by eGFR) during treatment with sparsentan or irbesartan regardless of original treatment arm randomization. These data should provide further insight into the role of proteinuria remission as indicating patient benefit through improved kidney survival in interventional trials in IgA nephropathy.

## Methods

### Study Design and Participants within the Double-Blind Period

PROTECT (NCT03762850) was a phase 3, randomized, active-controlled, double-blind, parallel-group, international, multicenter clinical trial designed to evaluate the efficacy and safety of sparsentan versus the maximum labeled dose of irbesartan in adults with IgA nephropathy who continued to have proteinuria despite maximized treatment with angiotensin-converting enzyme inhibitors or angiotensin II receptor blockers (ARBs). Study design and enrollment criteria have been previously reported.^10,11^ In brief, the trial was conducted at 134 sites in 18 countries including the United States and across Europe and the Asia Pacific region. Participant inclusion criteria were age at least 18 years with biopsy-proven primary IgA nephropathy, 24-hour proteinuria at least 1.0 g/d, eGFR at least 30 ml/min per 1.73 m^2^, systolic BP at or below 150 mm Hg, diastolic BP at or below 100 mm Hg, and stable angiotensin-converting enzyme inhibitor or ARB therapy for at least 12 weeks before screening at the patient's maximum tolerated dose, which was at least half of the maximum labeled dose. Participants were enrolled after institutional review board or ethics committee approvals at each investigational site in accordance with Good Clinical Practice and the Declaration of Helsinki. All participants provided written informed consent before study enrollment. The primary efficacy end point (change from baseline [BL] in urine protein-to-creatinine ratio [UPCR] based on a 24-hour urine sample at week 36), secondary efficacy end points (chronic eGFR slope [rate of eGFR change over weeks 6–110], total eGFR slope [day 1–week 110], change from BL in eGFR and proteinuria up to week 110, and proportion of patients reaching a composite kidney end point [defined below]), and safety end points of the PROTECT trial have been previously reported.^9,10^

### End Points

The main goal of this *post hoc* analysis was to compare the rate of decline in kidney function in patients who achieved CR through week 36 (CR36) or at any time up to week 110 (CR110) versus those who did not achieve this end point (non-CR), regardless of original randomization allocation. CR was defined as achieving 24-hour UPE of below 0.3 g/d. Absolute change in eGFR from BL to the end of the treatment period and eGFR chronic and total slopes were compared between CR and non-CR patients. In addition, an analysis of total eGFR slope, stratified by patient populations achieving UPE thresholds (below 0.3, 0.3 to <0.5, 0.5 to <1.0, and at least 1.0 g/d) at weeks 36 and 110, is presented.

Changes in proteinuria (UPCR and UPE), albuminuria (urine albumin-to-creatinine ratio [UACR]), and BP from BL during follow-up, proportion of patients who reached the composite kidney end point (defined as a confirmed 40% eGFR decline, kidney failure or initiation of KRT, or mortality due to any cause), and treatment-emergent adverse events (TEAEs) were additional end points and were compared in CR versus non-CR participants.

### Statistical Analysis Methods

Efficacy analyses and safety analyses were based on the full analysis set and safety analysis set, respectively, both of which were defined as all patients who were randomized and received at least one dose of randomized study drug. Pharmacokinetic (PK) analyses were based on the PK analysis set, which was defined as all patients who received at least one dose of study medication and had at least one confirmed, fasted, and analyzable sample. All statistical analyses were performed using SAS (version 9.4 or later).

eGFR slope was analyzed using a mixed model random coefficients analysis with indicator for CR at any time up to week 110, BL eGFR, analysis visit, CR-by-analysis visit, and randomization stratification factors as fixed effects; an unstructured covariance structure was used. A random intercept and random slope were included for each patient. The slope estimates, differences in slopes, and 95% confidence interval (CI) were extracted from the model; slopes and differences in slopes were annualized for ease of presentation and interpretation.

Change from BL in eGFR at week 6 and up to week 110 was analyzed through a mixed model for repeated measures (MMRMs) with CR versus no-CR at any time up to week 110, BL eGFR, analysis visit, CR-by-analysis visit interaction, and randomization stratification factors as fixed effects and patient as a random effect; an unstructured covariance structure was used. The analysis included CR status as a fixed patient characteristic to estimate the association between CR at any time and eGFR change. The least-squares (LSs) means, effect estimate (CR versus no-CR), and 95% CI were extracted from the model. The same model was applied to determine eGFR total slope by achieved UPE threshold of <0.3, 0.3 to <0.5, 0.5 to <1.0 and at least 1.0 g/d at weeks 36 and 110.

Change from BL in UPCR, UPE, and UACR at week 6 and up to week 110 was analyzed through an MMRM based on natural log (change from BL) quantitative urinalysis values with CR versus no-CR at any time up to week 110, log BL value, analysis visit, CR-by-analysis visit interaction, and randomization stratification factors as fixed effects and patient as a random effect; an unstructured covariance was used. Estimated LS means and 95% CIs were back-transformed to a ratio scale.

Change from BL in BP up to week 110 was analyzed through an MMRM with CR, BL value, analysis visit, CR-by-analysis visit interaction, and randomization stratification factors as fixed effects and patient as a random effect; an unstructured covariance was used. The LS means, effect estimate (CR versus no-CR), and 95% CI were extracted from the model.

The composite kidney end point was analyzed with Kaplan-Meier methodology without causal inference, censoring at study discontinuation within the double-blind period.

eGFR slope, change in eGFR, UACR, and BP, and composite kidney end point were analyzed using on-treatment data.^9^ TEAEs were coded using the Medical Dictionary for Regulatory Activities v23.0 and analyzed descriptively.

## Results

### BL Characteristics and Association with CR

Of 404 patients who were randomized and received study drug, 43 (11%) achieved CR at ≥1 visit by week 36 (CR36) and 361 (89%) did not (non-CR36); 85 (21%) patients achieved CR110 and 319 (79%) never achieved CR (non-CR110). Table 1 presents BL characteristics of CR and non-CR patients. Compared with non-CR patients, CR36 and CR110 achievers had lower BL proteinuria and higher BL eGFR. BL sex and race were similar across groups. Of the 43 patients who achieved CR36, 81% had received treatment with sparsentan compared with 19% who received irbesartan. Similarly, of the 85 patients who achieved CR110, 73% had received treatment with sparsentan compared with 27% who received irbesartan. The median time to first CR was 38.1 weeks (interquartile range, 12.9–70.1). Of the 85 patients who achieved CR110, UPE levels later rose to more than 0.75 g/d (at least one visit) in 30 (35%; median time to UPE >0.75 g/d: 24.2 weeks [interquartile range, 12.0–36.0]) but did not in 55 (65%).

**Table 1:** Demographics and baseline characteristics

Characteristics	All Patients (*N*=404) (Full Analysis Set)		All Patients (*N*=404) (Full Analysis Set)	
	CR (UPE <0.3 g/d) Achieved through Week 36		CR (UPE <0.3 g/d) Achieved at Any Time Up to Week 110	
	Yes (*n*=43)	No (*n*=361)	Yes (*n*=85)	No (*n*=319)
**Demographics and BL characteristics**				
Age, yr, mean (SD)	41 (12)	47 (12)	44 (14)	47 (12)
Sex, male, *n* (%)	32 (74)	250 (69)	54 (64)	228 (71)
**Race, *n* (%)**				
American Indian or Alaskan native	0 (0)	0 (0)	0 (0)	0 (0)
Asian	12 (28)	103 (29)	29 (34)	86 (27)
Black or African American	0 (0)	4 (1)	1 (1)	3 (1)
Native Hawaiian or other Pacific Islander	0 (0)	1 (<1)	0 (0)	1 (<1)
Other	2 (5)	11 (3)	2 (2)	11 (3)
White	29 (67)	243 (67)	53 (62)	219 (69)
Time from initial biopsy to informed consent, yr, median (IQR)	3.0 (1.0–6.0)	4.0 (1.0–10.0)	3.0 (1.0–7.0)	4.0 (1.0–10.0)
eGFR, ml/min per 1.73 m^2^, mean (SD)	67 (25)	56 (24)	65 (26)	55 (23)
UPE, g/d, median (IQR)	1.2 (1.0–1.6)	1.9 (1.3–2.9)	1.3 (1.0–1.9)	2.0 (1.4–3.0)
UPCR, g/g, median (IQR)	0.7 (0.5–1.1)	1.3 (0.9–1.8)	0.9 (0.7–1.2)	1.3 (0.9–1.9)
**BP, mm Hg, mean (SD)**				
Systolic	127.6 (13.6)	129.1 (13.5)	128.2 (14.8)	129.2 (13.1)
Diastolic	80.6 (10.1)	82.7 (10.7)	80.9 (11.1)	82.9 (10.5)
**Treatment assignment, *n* (%)**				
Sparsentan	35 (81)	167 (46)	62 (73)	140 (44)
Irbesartan	8 (19)	194 (54)	23 (27)	179 (56)

BL, baseline; CR, complete remission of proteinuria; IQR, interquartile range; UPCR, urine protein-to-creatinine ratio; UPE, urine protein excretion.

### Proteinuria Reduction

**Figure 1:**
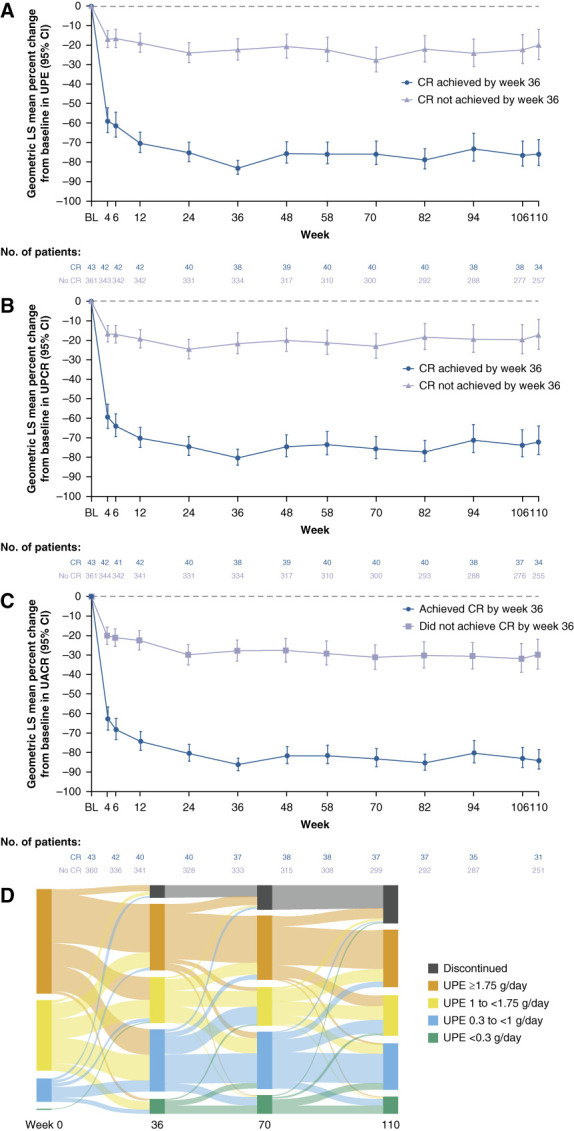
**Patients who achieved CR at any time by week 36 demonstrated greater and rapid reductions in proteinuria and albuminuria than those who did not.** Percent change from BL is shown for (A) UPE, (B) UPCR, and (C) UACR at each study visit. Sankey plot shows proteinuria levels throughout the study period* (D). BL, baseline; CI, confidence interval; CR, complete remission of proteinuria; LS, least-square; UACR, urine albumin-to-creatinine ratio; UPCR, urine protein-to-creatinine ratio; UPE, urine protein excretion.

**Figure 2:**
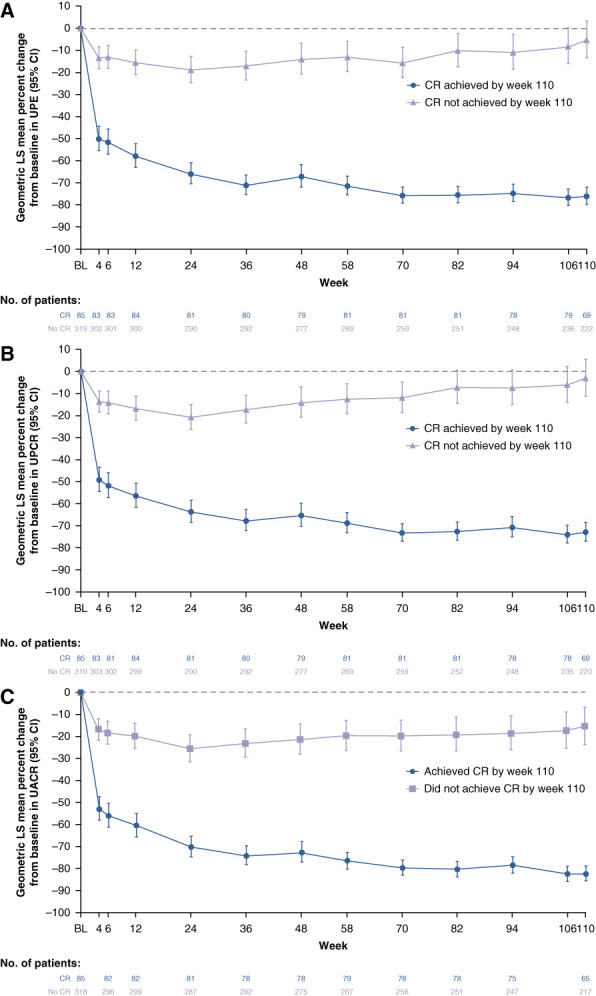
**Patients who achieved CR at any time up to week 110 demonstrated greater and rapid reductions in proteinuria and albuminuria than those who did not.** Percent change from BL is shown for (A) UPE, (B) UPCR, and (C) UACR at each study.

Compared with non-CR patients, CR36 and CR110 patients demonstrated a substantially greater and rapid reduction in UPE, observed already after 6 weeks of treatment, which was sustained through study follow-up (Figures 1A and 2A). Similar trends in UPCR (Figures 1B and 2B) and UACR (Figures 1C and 2C) were observed in CR36 or CR110 patients versus non-CR patients. Percent change from BL in proteinuria at weeks 6 and 110 is reported in Table 2. A Sankey plot visualization shows that approximately half of patients achieving CR36 continued to experience CR at weeks 70 and 110 (Figure 1D). A trend of sustained proteinuria reduction throughout follow-up was observed across all BL proteinuria categories (Figure 1D). Premature study drug discontinuation was less frequent in patients who achieved CR36 or CR110 compared with non-CR patients, mainly due to fewer discontinuations due to adverse events (AEs) and patient or physician decision (Table 3).

**Table 2:** Percent change from baseline in proteinuria at weeks 6 and 110^a^

Change in Proteinuria	All Patients (*N*=404) (Full Analysis Set)		All Patients (*N*=404) (Full Analysis Set)	
	CR (UPE <0.3 g/d) Achieved through Week 36		CR (UPE <0.3 g/d) Achieved at Any Time up to Week 110	
	Yes (*n*=43)	No (*n*=361)	Yes (*n*=85)	No (*n*=319)
**Change in UPE at week 6, LS mean (95% CI), %**	−61 (−67 to −54)	−17 (−21 to −12)	−52 (−57 to −46)	−13 (−18 to −8)
Ratio (CR versus no-CR), (95% CI; *P* value)	0.46 (0.39 to 0.55; <0.001)		0.56 (0.49 to 0.64; <0.001)	
**Change in UPE at week 110, LS mean (95% CI), %**	−76 (−82 to −69)	−20 (−27 to −12)	−76 (−80 to −72)	−5 (−13 to 3)
Ratio (CR versus no-CR), (95% CI; *P* value)	0.30 (0.22 to 0.40; <0.001)		0.25 (0.21 to 0.30; <0.001)	
**Change in UPCR at week 6, LS mean (95% CI), %**	−64 (−69 to −58)	−17 (−21 to −12)	−52 (−57 to −46)	−14 (−19 to −9)
Ratio (CR versus no-CR), (95% CI; *P* value)	0.43 (0.37 to 0.51; <0.001)		0.56 (0.49 to 0.64; <0.001)	
**Change in UPCR at week 110, LS mean (95% CI), %**	−72 (−79 to −64)	−17 (−25 to −9)	−73 (−77 to −68)	−3 (−11 to 6)
Ratio (CR versus no-CR), (95% CI; *P* value)	0.34 (0.25 to 0.44; <0.001)		0.28 (0.23 to 0.33; <0.001)	
**Change in UACR at week 6, LS mean (95% CI), %**	−68 (−73 to −63)	−21 (−25 to −16)	−56 (−61 to −50)	−18 (−23 to −13)
Ratio (CR versus no-CR), (95% CI; *P* value)	0.40 (0.34 to 0.48; <0.001)		0.54 (0.47 to 0.62; <0.001)	
**Change in UACR at week 110, LS mean (95% CI), %**	−84 (−88 to −78)	−30 (−37 to −22)	−83 (−86 to −79)	−15 (−23 to −7)
Ratio (CR versus no-CR), (95% CI; *P* value)	0.23 (0.16 to 0.31; <0.001)		0.21 (0.17 to 0.25; <0.001)	

CI, confidence interval; CR, complete remission of proteinuria; LS, least square; UACR, urine albumin-to-creatinine ratio; UPCR, urine protein-to-creatinine ratio; UPE, urine protein excretion.

a Changes are from baseline and geometric LS mean. *P* values are nominal.

**Table 3:** Patient disposition

*n* (%)	All Patients (*N*=404) (Full Analysis Set)		All Patients (*N*=404) (Full Analysis Set)	
	CR (UPE <0.3 g/d) Achieved through Week 36		CR (UPE <0.3 g/d) Achieved at Any Time Up to Week 110	
	Yes (*n*=43)	No (*n*=361)	Yes (*n*=85)	No (*n*=319)
Completed treatment in the double-blind period	39 (91)	289 (80)	80 (94)	248 (78)
Discontinued treatment prematurely in the double-blind period	4 (9)	72 (20)	5 (6)	71 (22)
Reasons for premature treatment discontinuations				
*AEs*	3 (7)	34 (9)	3 (4)	34 (11)
*Patient decision*	1 (2)	25 (7)	2 (2)	24 (8)
*Physician decision*	0 (0)	7 (2)	0 (0)	7 (2)
*Pregnancy*	0 (0)	2 (1)	0 (0)	2 (1)
*Protocol deviation*	0 (0)	2 (1)	0 (0)	2 (1)
*Receipt of kidney transplant or initiation of chronic dialysis*	0 (0)	2 (1)	0 (0)	2 (1)

AE, adverse event; CR, complete remission of proteinuria; UPE, urine protein excretion.

### eGFR Trajectory and Composite Kidney End Points of Patients Who Did or Did Not Achieve CR

**Figure 3:**
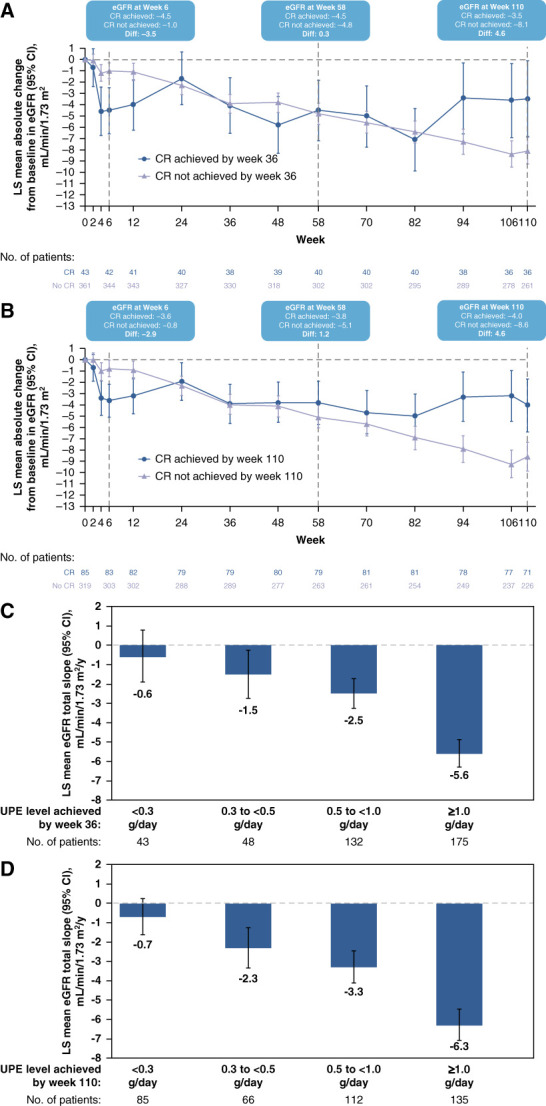
**Patients who achieved lower proteinuria levels had better eGFR preservation than those who did not.** Figures show absolute change from BL in eGFR at each study visit in patients who did or did not achieve (A) CR36* or (B) CR110† and eGFR rate of change (total slope) by UPE threshold achieved at (C) week 36 or (D) week 110. Week 58 eGFR changes are presented (A and B), as they present the 1-year eGFR changes after the week-6 eGFR changes.

**Figure 4:**
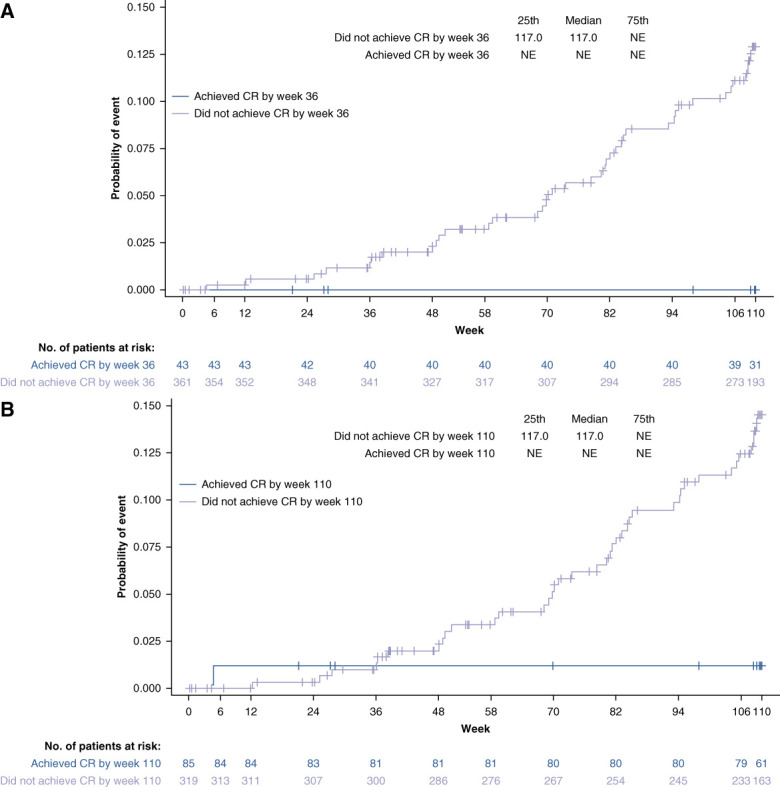
**Patients were less likely to reach the composite kidney end point if they achieved CR than if they did not.** Kaplan-Meier plots show time to reach the composite kidney end point in patients who did or did not achieve (A) CR36* and (B) CR110†. NE, not estimable.

After an initial acute reduction, CR36 and CR110 patients demonstrated stable eGFR compared with a sustained gradual reduction in eGFR over the follow-up period observed in non-CR patients (Figure 3, A and B). As presented in Table 4, the decline in eGFR was markedly slower in CR36 and CR110 patients compared with non-CR patients. Total eGFR slope in CR110 patients was slower versus non-CR patients (−0.7 [95% CI, −1.7 to 0.3] versus −4.2 [95% CI, −4.8 to −3.7] ml/min per 1.73 m^2^ per year; nominal *P* < 0.001). Similarly, the chronic eGFR slope in CR110 patients was slower than in non-CR patients (−0.4 [95% CI, −1.4 to 0.6] versus −4.2 [95% CI, −4.7 to −3.7] ml/min per 1.73 m^2^ per year; nominal *P* < 0.001). Further stratification of proteinuria remission status by using achieved UPE thresholds of <0.3, 0.3 to <0.5, 0.5 to <1.0, and at least 1.0 g/d at weeks 36 and 110 showed similar effects as our main analysis with progressively higher rates of eGFR decline in higher UPE subgroups (Figure 3, C and D). The composite kidney end point did not occur in CR36 patients and occurred less often among CR110 patients (*n*=1 [1%]) compared with those who never achieved CR36 (*n*=44 [12%]) or CR110 (*n*=43 [14%]; Figure 4).

**Table 4:** Change in eGFR by study visit and rate of change over time^a^

Rate of Change in eGFR	All Patients (*N*=404) (Full Analysis Set)		All Patients (*N*=404) (Full Analysis Set)	
	CR (UPE <0.3 g/d) Achieved through Week 36		CR (UPE <0.3 g/d) Achieved at Any Time Up to Week 110	
	Yes (*n*=43)	No (*n*=361)	Yes (*n*=85)	No (*n*=319)
**Change from BL in eGFR at week 6, LS mean (95% CI), ml/min per 1.73 m** ^**2**^	−4.5 (−6.59 to −2.50)	−1.0 (−1.70 to −0.30)	−3.6 (−5.10 to −2.17)	−0.8 (−1.52 to −0.02)
Difference (95% CI; *P* value)	−3.5 (−5.72 to −1.37; =0.001)		−2.9 (−4.53 to −1.20; <0.001)	
**Change from BL in eGFR at week 110, LS mean (95% CI), ml/min per 1.73 m** ^**2**^	−3.5 (−6.81 to −0.11)	−8.1 (−9.28 to −6.89)	−4.0 (−6.40 to −1.69)	−8.6 (−9.90 to −7.34)
Difference (95% CI; *P* value)	4.6 (1.06 to 8.18; =0.01)		4.6 (1.88 to 7.27; <0.001)	
**eGFR total slope, LS mean (95% CI), ml/min per 1.73 m**^**2**^ **per year**	−0.6 (−2.00 to 0.85)	−3.8 (−4.30 to −3.29)	−0.7 (−1.65 to 0.29)	−4.2 (−4.77 to −3.72)
Difference (95% CI; *P* value)	3.2 (1.71 to 4.74; <0.001)		3.6 (2.46 to 4.67; <0.001)	
**eGFR chronic slope, LS mean (95% CI), ml/min per 1.73 m**^**2**^ **per year**	−0.2 (−1.67 to 1.21)	−3.7 (−4.24 to −3.21)	−0.4 (−1.35 to 0.60)	−4.2 (−4.74 to −3.67)
Difference (95% CI; *P* value)	3.5 (1.97 to 5.03; <0.001)		3.8 (2.71 to 4.94; <0.001)	

BL, baseline; CI, confidence interval; CR, complete remission of proteinuria; LS, least square; UPE, urine protein excretion.

a *P* values are nominal.

### BP in Patients Who Did or Did Not Achieve CR

**Figure 5:**
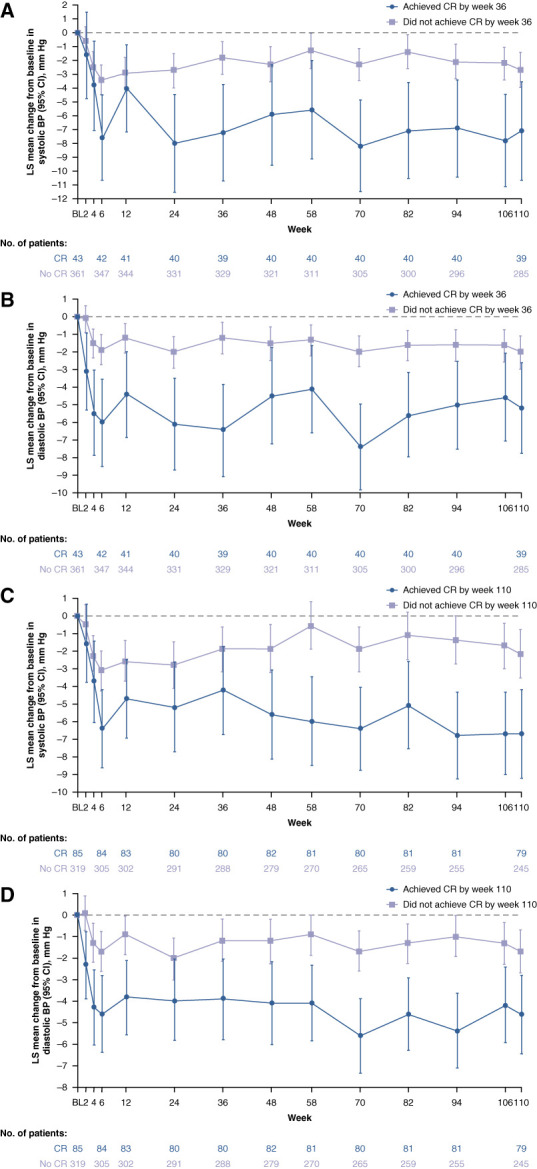
**Systolic and diastolic BP showed larger acute reductions, followed by relative stability, in patients who achieved CR than in those who did not.** Absolute change from BL in BP at each study visit is shown in patients who did or did not achieve CR36 (A and B) and CR110 (C and D)*.

Systolic and diastolic BP each showed larger acute reductions in CR36 and CR110 patients compared with non-CR patients. After week 6, BP levels remained relatively stable through week 110 (Figure 5). For systolic and diastolic BP, the difference between CR and non-CR patients oscillated around 4 mm Hg.

### PK

As presented in Table 5, at week 6, CR patients had similar blood concentrations of sparsentan compared with non-CR patients, which was sustained through the study follow-up.

**Table 5:** Trough pharmacokinetic concentrations at weeks 6 and 110 in patients who did or did not achieve complete remission of proteinuria at any time by week 110^a^

Trough PK Concentration (ng/ml)	Irbesartan		Sparsentan	
	CR at Any Time on Treatment (*n*=23)	No CR at Any Time on Treatment (*n*=166)	CR at Any Time on Treatment (*n*=61)	No CR at Any Time on Treatment (*n*=136)
**Week 6, *n***	22	154	59	129
Mean (SD)	444.2 (382.57)	443.7 (651.22)	2024.5 (1916.70)	1911.5 (2034.91)
SEM	81.56	52.48	249.53	179.16
Median	345.5	254.5	1380.0	1250.0
*IQR*	133.0–669.0	134.0–498.0	694.0–2880.0	695.0–2060.0
Min, max	56, 1440	6, 4870	21, 9030	82, 10,700
Geometric mean	303.2	252.4	1214.6	1217.3
Geometric CV (%)	118	146	179	131
No. of BLQs	0	5	0	3
**Week 110, *n***	19	121	57	111
Mean (SD)	525.4 (1054.99)	494.2 (676.94)	2224.6 (2456.99)	1807.3 (1863.69)
SEM	242.03	61.54	325.44	176.89
Median	195.0	270.0	1290.0	1290.0
*IQR*	121.0–528.0	153.0–518.0	781.0–2350.0	714.0–2190.0
Min, max	63, 4780	8, 3960	15, 10,800	71, 12,100
Geometric mean	249.2	268.2	1288.8	1202.5
Geometric CV (%)	147	163	173	119
No. of BLQs	0	10	0	5

BLQ, below lower limit of quantification; CR, complete remission of proteinuria; CR110, complete remission of proteinuria achieved at any time by week 110; CV, coefficient of variation; IQR, interquartile range; PK, pharmacokinetics.

a Pharmacokinetics analyses were based on the pharmacokinetics analysis set, which was defined as all patients who received at least one dose of study medication and have at least one confirmed, fasted, and analyzable sample.

### Safety in Patients Who Did or Did Not Achieve CR110

TEAEs were reported in 79 (93%) of 85 CR110 patients and 285 (89%) of 319 non-CR110 patients (Table 6). TEAEs that were reported more frequently (over 5 percentage points) in CR110 patients versus non-CR110 patients were coronavirus disease 2019 and headache. Several TEAEs were reported more frequently (over 5 percentage points) in non-CR110 patients versus CR110 patients: hypertension, proteinuria, and CKD.

**Table 6:** Treatment-emergent adverse events^a^

*n (%)*	All Patients (*N*=404) (Safety Analysis Set)	
	CR (UPE <0.3 g/d) Achieved at Any Time Up to Week 110	
	Yes (*n*=85)	No (*n*=319)
Any TEAE	79 (93)	285 (89)
**TEAEs in ≥5% of patients in the CR group or ≥5% of patients in the non-CR group**		
COVID-19	28 (33)	71 (22)
Headache	16 (19)	37 (12)
Hyperkalemia	13 (15)	45 (14)
Edema peripheral	12 (14)	43 (13)
Dizziness	11 (13)	32 (10)
Hypotension	10 (12)	24 (8)
Diarrhea	8 (9)	21 (7)
Upper respiratory tract infection	6 (7)	30 (9)
Arthralgia	6 (7)	21 (7)
Back pain	6 (7)	22 (7)
Blood creatine phosphokinase increased	6 (7)	19 (6)
Gout	6 (7)	15 (5)
Myalgia	6 (7)	8 (3)
Hypertension	5 (6)	45 (14)
Muscle spasms	5 (6)	26 (8)
Nasopharyngitis	5 (6)	26 (8)
Fatigue	5 (6)	23 (7)
Urinary tract infection	5 (6)	14 (4)
AKI	5 (6)	12 (4)
Hematuria	5 (6)	8 (3)
Gastroenteritis	5 (6)	7 (2)
Anxiety	5 (6)	4 (1)
Anemia	4 (5)	21 (7)
Renal impairment	4 (5)	15 (5)
Alanine aminotransferase increased	4 (5)	14 (4)
Gastroesophageal reflux disease	4 (5)	14 (4)
Abdominal pain	4 (5)	12 (4)
Nausea	4 (5)	11 (3)
Aspartate aminotransferase increased	4 (5)	9 (3)
Orthostatic hypotension	4 (5)	9 (3)
Osteoarthritis	4 (5)	5 (2)
Pruritus	3 (4)	16 (5)
Hyperuricemia	3 (4)	15 (5)
Vertigo	3 (1)	4 (5)
Blood creatinine increased	2 (2)	22 (7)
Cough	2 (2)	20 (6)
Pain in extremity	2 (2)	16 (5)
Dyspepsia	2 (2)	15 (5)
Proteinuria	1 (1)	27 (8)
Lipase increased	1 (1)	20 (6)
CKD	0 (0)	18 (6)
Asthenia	0 (0)	15 (5)
**TEAEs of special interest**		
Any hypotension-associated TEAEs	14 (16)	32 (10)
*Hypotension*	10 (12)	24 (8)
*Orthostatic hypotension*	4 (5)	9 (3)
*BP systolic decreased*	0 (0)	1 (<1)
Patients with any fluid retention-associated TEAEs	17 (20)	50 (16)
*Edema peripheral*	12 (14)	43 (13)
*Peripheral swelling*	3 (4)	5 (2)
*Joint swelling*	2 (2)	4 (1)
*Fluid overload*	0 (0)	1 (<1)
*Lymphedema*	0 (0)	1 (<1)
*Edema*	0 (0)	1 (<1)
*Perinephric collection*	0 (0)	1 (<1)
*Pleural effusion*	1 (1)	0 (0)
Patients with any anemia-associated TEAEs	4 (5)	28 (9)
*Anemia*	4 (5)	21 (7)
*Iron deficiency anemia*	0 (0)	4 (1)
*Hemoglobin decreased*	0 (0)	2 (1)
*Macrocytosis*	0 (0)	2 (1)
Patients with any hyperkalemia-associated TEAEs	14 (16)	47 (15)
*Hyperkalemia*	13 (15)	45 (14)
*Blood potassium increased*	1 (1)	3 (1)
Patients who discontinued treatment due to TEAEs	3 (4)	34 (11)

COVID-19, coronavirus disease 2019; CR, complete remission of proteinuria; TEAE, treatment-emergent adverse event; UPE, urine protein excretion.

a Based on the safety analysis set.

Patients who achieved CR110 were more likely to experience TEAEs associated with hypotension (hypotension, orthostatic hypotension, or BP systolic decreased) versus non-CR110 patients; by contrast, patients who achieved CR110 were also less likely to experience hypertension. TEAEs related to hyperkalemia and fluid retention were similar between groups, as were TEAEs of alanine aminotransferase increased and aspartate aminotransferase increased. The proportion of patients who discontinued treatment due to AEs was higher in non-CR110 patients than in CR110 patients (Table 6).

## Discussion

This *post hoc* analysis from the PROTECT trial demonstrated that participants who achieved CR experienced less eGFR decline and fewer clinical kidney end points compared with participants who did not achieve CR. CR was achieved more frequently in the sparsentan group compared with the irbesartan group, consistent with previous results showing the more pronounced proteinuria lowering efficacy of sparsentan compared with maximized dose of irbesartan.^9,10^ CR was associated with a greater reduction in BP; however, CR was not associated with an increased risk of edema, anemia, or hyperkalemia (safety issues that are potentially related to the mechanism of action of the study drugs). This suggests that a strong proteinuria reduction with these study drugs does not necessarily lead to an increased risk of AEs.^9,12^

Proteinuria is a well-established risk marker of adverse kidney outcomes in patients with IgA nephropathy.^13^ Observational studies demonstrated a strong and consistent association between proteinuria and progressive eGFR decline.^2,3,5,7^ Experimental studies reported that increased tubular albumin exposure stimulates pro-inflammatory pathways leading to tubulointerstitial damage and nephron loss indicating that a biologic plausible mechanism between increased glomerular albumin leakage and kidney damage exists.^14^ Earlier studies also demonstrated that a proteinuria reduction with therapeutic interventions to levels <0.3 g/d is associated with a marked stabilization of eGFR over time,^3^ which has been confirmed in more recent studies.^4,6^ The current analyses confirm and extend these findings to a contemporary global IgA nephropathy population treated according to optimal care and studied in a rigorous clinical trial setting. Accordingly, the Kidney Disease Improving Global Outcomes (KDIGO) guidelines recommend that proteinuria should be sustained to levels <0.5 g/d, ideally <0.3 g/d, and that the rate of kidney function decline be <1 ml/min per 1.73 m^2^ per year.^15^ The current data from the PROTECT trial support this recommendation as patients who achieved CR36 or CR110 had a marked stabilization of eGFR and reduced risk of reaching the composite kidney end point. Moreover, a gradual steeper eGFR decline was observed in participants who achieved UPE thresholds at week 36 or 110 of <0.3, 0.3 to <0.5, 0.5 to < 1.0, and at or more than 1.0 g/d, supporting the 2025 KDIGO guidelines recommendation.^15^ While CR was achieved more frequently with sparsentan versus irbesartan, this association between CR achievement and kidney function preservation in this treatment-agnostic analysis suggests that achieving CR is an important treatment goal irrespective of whether patients receive RAS-inhibition alone or DEARA.

Reaching the composite kidney end point was rare among participants who achieved CR, consistent with the preservation of eGFR over time in these patients. This finding highlights the strong association between proteinuria reduction and prevention of kidney failure and provides additional support for proteinuria change as a reasonably likely surrogate outcome in patients with IgA nephropathy. However, these analyses alone do not provide evidence about a causal link between achieving CR and reducing kidney failure. Specifically, although we adjusted our analyses for multiple potential confounders, there may have been unmeasured differences in BL characteristics between participants who did or did not achieve CR, which were not accounted for in the statistical analyses. Residual confounding therefore cannot be excluded. In addition, CR was paralleled by a greater reduction in BP, which may also contribute to kidney protection. To address the question of whether proteinuria change is a reasonably likely surrogate for kidney failure and a potential target for treatment, trial-level analyses have been performed and showed strong associations between treatment effects on early proteinuria change with treatment effects on eGFR decline or clinical outcomes.^16^ As a result, early change in proteinuria is now used as a surrogate outcome for conditional drug regulatory approval in clinical trials of IgA nephropathy.^7,16^ Our analyses confirm this growing body of evidence and add new data from a randomized controlled trial on the relationship between proteinuria reduction and kidney failure during guideline-recommended treatment with ARB or DEARA treatment.

Understanding the phenotype of patients who achieved CR is clinically important. Participants who achieved CR were younger, had higher BL eGFR, lower BP, and shorter time from initial kidney biopsy to informed consent compared with those who did not achieve CR. This suggests that participants who achieved CR may have less advanced disease, and possibly a preserved endothelial function with less arterial stiffness, making them more responsive to RAS-inhibition or DEARA treatment. Proteinuria levels at BL were also lower in participants who achieved CR, which may be a reflection of a more preserved vascular system but could also simply be a result of the definition for CR as the threshold of 0.3 g/d can be more easily achieved in participants with lower proteinuria levels at study entry. Notably, some patients had a BL proteinuria level of <1.0 g/d, as shown in the Sankey plot (Figure 1D). This probably reflects random variability in proteinuria, as these patients qualified for study participation based on their screening proteinuria levels of >1.0 g/d. Nevertheless, participants who achieved CR experienced a greater percent reduction from BL in proteinuria compared with those not achieving CR indicating that they showed a substantial pharmacotherapeutic response to either irbesartan or sparsentan.

Participants who achieved CR experienced a larger acute reduction in eGFR at week 6 compared with those who did not achieve CR. During subsequent follow-up, eGFR decline was significantly less in participants who achieved CR compared with those who did not achieve CR. This pattern is reminiscent of other drugs that exert hemodynamic effects and likely reflects a reduction in intraglomerular pressure.^17^ The greater reduction in BP among participants who achieved CR suggests a hemodynamic influence. However, several observations suggest that the reduction in intraglomerular pressure is likely not the only factor accounting for the reduction in proteinuria. First, the profound reduction in proteinuria upon sparsentan initiation seems out of proportion compared to the relatively modest reduction of circa 1.2 ml/min per 1.73 m^2^ in eGFR.^9^ Furthermore, the reduction in eGFR was fully present after 4 weeks after which eGFR stabilized or even slightly increased. By contrast, proteinuria continued to decline after 6 weeks, reached a maximal effect after 36 weeks, and was maintained thereafter. A spectrum of other potential mechanisms (based on experimental evidence) by which sparsentan can reduce proteinuria has been recently reviewed.^12^

Participants who achieved CR did not experience more peripheral edema, anemia, or hyperkalemia compared with participants who did not achieve CR. This may be attributed to a difference in efficacy and safety exposure-response relationships. Indeed, previous studies demonstrated a dissociation between the albuminuria and fluid retention responses to the endothelin receptor antagonist atrasentan.^18^ Similarly, patients who achieved a substantial albuminuria reduction with ARBs did not experience hyperkalemia more frequently compared with patients without a reduction in albuminuria.^19^ These observations have direct clinical implication since they suggest that achieving CR is not limited by higher risk of side effects and that the proteinuria lowering efficacy of sparsentan or irbesartan does not correlate with or depend upon any degree of fluid retention or hyperkalemia.

The most obvious study limitation is that this was a *post hoc* exploratory analysis of a randomized controlled trial and is therefore prone to chance findings. We also did not adjust for time-varying changes in rescue immunosuppressive medication initiation; however, only 22 (5%) participants started these medications during follow-up,^9^ and the effect on the results is therefore considered minimal. Twenty-seven percent of patients in the irbesartan arm eventually achieved delayed remission, suggesting that continued RAS-inhibition may still yield benefit over time. Finally, we studied a cohort enrolled in a clinical trial and selected by virtue of the inclusion and exclusion criteria and other factors that determine trial participation.^9^ The results, although consistent with cohort studies on this topic and confirmatory in that regard, can therefore not be generalized to patients with IgA nephropathy who do not share the inclusion and exclusion criteria of PROTECT.

In conclusion, participants in PROTECT who achieved CR36 or CR110 showed a greater preservation of eGFR and less kidney failure compared with non-CR participants. Considerably more participants in the sparsentan group achieved CR compared with the irbesartan group. Overall, these data reinforce the KDIGO guideline recommendations to maintain proteinuria levels in IgA nephropathy ideally <0.3 g/d.^15^

## Supplementary Material

## Data Availability

Original data generated for the study will be made available upon reasonable request to the corresponding author. Data Type: Clinical Trial Data. Reason for Restricted Access: Travere is committed to data transparency and sharing data collected in completed and published phase 3 clinical trials, observational trials, and post-marketing studies to further research while ensuring that patient privacy is protected. Pertinent individual patient-level data that underlie the results reported in a manuscript may be made available after deidentification. Relevant information may include redacted study protocol and redacted clinical study report. Requests for clinical trial data, including language stating its intended use, should be directed to datarequest@travere.com. If approved, the requested information will be provided to the requestor after signing a data access agreement. Requests can be made following completion of the study and full publication of the study data in a peer reviewed journal for up to 36 months following its publication. Travere reserves the right to decline or recommend modifications to a request if it does not comply with the data sharing policy or if it is determined that the request is made by a biased source.

## Declarative Statements

This study includes clinical experimentation and received Institutional Review Board or Ethics Committee approval. All patients provided written informed consent. This study includes clinical experimentation and complies with the Declaration of Helsinki. The clinical and research activities being reported are consistent with the Principles of the Declaration of Istanbul as outlined in the Declaration of Istanbul on Organ Trafficking and Transplant Tourism.
