# Supplementary material for: Association between Complete Proteinuria Remission and Kidney Function in the Phase 3 PROTECT Trial of Sparsentan in IgA Nephropathy
Source: Clin J Am Soc Nephrol. 2025 Dec 22;21(4):578–92. doi: 10.2215/CJN.0000000961 (PMC13065159; doi:10.2215/CJN.0000000961)
Supplement: Supplementary file 1 [file cjasn-21-578-s001.pdf]

ASN American Society of Nephrology JASN Journal of the American Society of Nephrology KIDNEY360 Accessing Our World From Every Angle CJASN Clinical Journal of the American Society of Nephrology

ASN Journal Disclosure Form

As per ASN journal policy, I have disclosed any financial relationships or commitments I have held in the past 36 months as included below. I have listed my Current Employer below to indicate there is a relationship requiring disclosure. If no relationship exists, my Current Employer is not listed.

H. Heerspink reports the following:
Employer: University Medical Center Groningen; Consultancy: Ongoing consultancy agreements with AstraZeneca, Alexion, Bayer, Boehringer Ingelheim, Biocity Therapeutic, Dimerix, Eli-Lilly, Gilead, Janssen, Novartis, NovoNordisk, Roche, Travere Therapeutics; Research Funding: AstraZeneca, Bayer, Boehringer Ingelheim, NovoNordisk and Janssen research support (grant funding directed to employer); Honoraria: Lecture fees from AstraZeneca and NovoNordisk; and Speakers Bureau: AstraZeneca, Bayer, Novo Nordisk.

I understand that the information above will be published within the journal article, if accepted, and that failure to comply and/or to accurately and completely report the potential financial conflicts of interest could lead to the following: 1) Prior to publication, article rejection, or 2) Post-publication, sanctions ranging from, but not limited to, issuing a correction, reporting the inaccurate information to the authors' institution, banning authors from submitting work to ASN journals for varying lengths of time, and/or retraction of the published work.

Name: Hiddo Jan L. Heerspink
Manuscript ID: CJASN-2025-001116R2
Manuscript Title: Kidney function outcomes with complete remission of proteinuria in the PROTECT study of sparsentan in IgA nephropathy
Date of Completion: October 15, 2025
Disclosure Updated Date: June 7, 2025

ASN American Society of Nephrology | JASN Journal of the American Society of Nephrology | KIDNEY360 Accessing Our World From Every Angle | CJASN Clinical Journal of the American Society of Nephrology

ASN Journal Disclosure Form

As per ASN journal policy, I have disclosed any financial relationships or commitments I have held in the past 36 months as included below. I have listed my Current Employer below to indicate there is a relationship requiring disclosure. If no relationship exists, my Current Employer is not listed.

B. Hendry reports the following:
Employer: Travere Therapeutics; Ownership Interest: Travere Therapeutics; Advisory or Leadership Role: South West Thames Institute for Renal Research (Board Chair);; and Other Interests or Relationships: Emeritus Professor of Medicine, Kings College London.

I understand that the information above will be published within the journal article, if accepted, and that failure to comply and/or to accurately and completely report the potential financial conflicts of interest could lead to the following: 1) Prior to publication, article rejection, or 2) Post-publication, sanctions ranging from, but not limited to, issuing a correction, reporting the inaccurate information to the authors' institution, banning authors from submitting work to ASN journals for varying lengths of time, and/or retraction of the published work.

Name: Bruce M. Hendry
Manuscript ID: CJASN-2025-001116R2
Manuscript Title: Kidney function outcomes with complete remission of proteinuria in the PROTECT study of sparsentan in IgA nephropathy
Date of Completion: October 16, 2025
Disclosure Updated Date: October 16, 2025

ASN American Society of Nephrology | JASN Journal of the American Society of Nephrology | KIDNEY360 Accessing Our World From Every Angle | CJASN Clinical Journal of the American Society of Nephrology

ASN Journal Disclosure Form

As per ASN journal policy, I have disclosed any financial relationships or commitments I have held in the past 36 months as included below. I have listed my Current Employer below to indicate there is a relationship requiring disclosure. If no relationship exists, my Current Employer is not listed.

R. Komers reports the following:
Employer: Travere Therapeutics; San Diego, CA ; 3611 Valley Centre Drive, Suite 300; San Diego, CA 92130;; Ownership Interest: Travere Therapeutics; and Patents or Royalties: Travere Therapeutics.

I understand that the information above will be published within the journal article, if accepted, and that failure to comply and/or to accurately and completely report the potential financial conflicts of interest could lead to the following: 1) Prior to publication, article rejection, or 2) Post-publication, sanctions ranging from, but not limited to, issuing a correction, reporting the inaccurate information to the authors' institution, banning authors from submitting work to ASN journals for varying lengths of time, and/or retraction of the published work.

Name: Radko Komers
Manuscript ID: CJASN-2025-001116R2
Manuscript Title: Kidney function outcomes with complete remission of proteinuria in the PROTECT study of sparsentan in IgA nephropathy
Date of Completion: October 14, 2025
Disclosure Updated Date: October 14, 2025

ASN American Society of Nephrology | JASN Journal of the American Society of Nephrology | KIDNEY360 Accessing Our World From Every Angle | CJASN Clinical Journal of the American Society of Nephrology

ASN Journal Disclosure Form

As per ASN journal policy, I have disclosed any financial relationships or commitments I have held in the past 36 months as included below. I have listed my Current Employer below to indicate there is a relationship requiring disclosure. If no relationship exists, my Current Employer is not listed.

A. Mercer reports the following:
Employer: JAMCO Pharma Consulting AB; Consultancy: Travere Therapeutics, Vera Therapeutics, HiBio/Biogen, Dimerix, Timberlyne Therapeutics, Advanz Pharma; and Ownership Interest: JAMCO Pharma Consulting AB.

I understand that the information above will be published within the journal article, if accepted, and that failure to comply and/or to accurately and completely report the potential financial conflicts of interest could lead to the following: 1) Prior to publication, article rejection, or 2) Post-publication, sanctions ranging from, but not limited to, issuing a correction, reporting the inaccurate information to the authors' institution, banning authors from submitting work to ASN journals for varying lengths of time, and/or retraction of the published work.

Name: Alex Mercer
Manuscript ID: CJASN-2025-001116R1
Manuscript Title: Kidney function outcomes with complete remission of proteinuria in the PROTECT study of sparsentan in IgA nephropathy
Date of Completion: September 16, 2025
Disclosure Updated Date: April 8, 2025

ASN
American Society of Nephrology
JASN
Journal of the American Society of Nephrology
KIDNEY360
Accessing Our World From Every Angle
CJASN
Clinical Journal of the American Society of Nephrology

ASN Journal Disclosure Form

As per ASN journal policy, I have disclosed any financial relationships or commitments I have held in the past 36 months as included below. I have listed my Current Employer below to indicate there is a relationship requiring disclosure. If no relationship exists, my Current Employer is not listed.

E. Murphy reports the following:
Employer: Travere Therapeutics, Inc.; and Ownership Interest: Travere Therapeutics, Inc.

I understand that the information above will be published within the journal article, if accepted, and that failure to comply and/or to accurately and completely report the potential financial conflicts of interest could lead to the following: 1) Prior to publication, article rejection, or 2) Post-publication, sanctions ranging from, but not limited to, issuing a correction, reporting the inaccurate information to the authors' institution, banning authors from submitting work to ASN journals for varying lengths of time, and/or retraction of the published work.

Name: Edward Murphy
Manuscript ID: CJASN-2025-001116R2
Manuscript Title: Kidney function outcomes with complete remission of proteinuria in the PROTECT study of sparsentan in IgA nephropathy."
Date of Completion: October 14, 2025
Disclosure Updated Date: October 14, 2025

ASN American Society of Nephrology
JASN Journal of the American Society of Nephrology
KIDNEY360 Accessing Our World From Every Angle
CJASN Clinical Journal of the American Society of Nephrology

ASN Journal Disclosure Form

As per ASN journal policy, I have disclosed any financial relationships or commitments I have held in the past 36 months as included below. I have listed my Current Employer below to indicate there is a relationship requiring disclosure. If no relationship exists, my Current Employer is not listed.

P. Preciado reports the following:
Employer: Travere Therapeutics Inc; Consultancy: PPR Life Science Consultant LLC; and Ownership Interest: Travere Therapeutics.

I understand that the information above will be published within the journal article, if accepted, and that failure to comply and/or to accurately and completely report the potential financial conflicts of interest could lead to the following: 1) Prior to publication, article rejection, or 2) Post-publication, sanctions ranging from, but not limited to, issuing a correction, reporting the inaccurate information to the authors' institution, banning authors from submitting work to ASN journals for varying lengths of time, and/or retraction of the published work.

Name: Priscila Preciado
Manuscript ID: CJASN-2025-001116R2
Manuscript Title: Kidney function outcomes with complete remission of proteinuria in the PROTECT study of sparsentan in IgA nephropathy
Date of Completion: October 15, 2025
Disclosure Updated Date: October 15, 2025

ASN American Society of Nephrology | JASN Journal of the American Society of Nephrology | KIDNEY360 Accessing Our World From Every Angle | CJASN Clinical Journal of the American Society of Nephrology

ASN Journal Disclosure Form

As per ASN journal policy, I have disclosed any financial relationships or commitments I have held in the past 36 months as included below. I have listed my Current Employer below to indicate there is a relationship requiring disclosure. If no relationship exists, my Current Employer is not listed.

B. Rovin reports the following:
Employer: Ohio State University Wexner Medical Center; Consultancy: Genentech, Alexion, Aurinia, Roche, Calliditas, BMS, Travere, Novartis, Biogen, GSK, Astra Zeneca, Otsuka, Artiva, Regeneron, Century, Vera, Vertex; Research Funding: Biogen; Honoraria: Genentech, Alexion, Aurinia, Roche, Calliditas, BMS, Travere, Novartis, Biogen, GSK, Astra Zeneca, Otsuka, Artiva, Regeneron, Century, Vera, Vertex; Advisory or Leadership Role: Kidney International, Lupus Foundation of America, UpToDate, KDIGO,; and Other Interests or Relationships: I do a lot of work with the ASN, mostly educational courses.; I work with the NKF and the ISN; I work with the LFA.

I understand that the information above will be published within the journal article, if accepted, and that failure to comply and/or to accurately and completely report the potential financial conflicts of interest could lead to the following: 1) Prior to publication, article rejection, or 2) Post-publication, sanctions ranging from, but not limited to, issuing a correction, reporting the inaccurate information to the authors' institution, banning authors from submitting work to ASN journals for varying lengths of time, and/or retraction of the published work.

Name: Brad Rovin
Manuscript ID: CJASN-2025-001116R2
Manuscript Title: Kidney function outcomes with complete remission of proteinuria in the PROTECT study of sparsentan in IgA nephropath
Date of Completion: October 13, 2025
Disclosure Updated Date: October 13, 2025

ASN American Society of Nephrology JASN Journal of the American Society of Nephrology KIDNEY360 Accessing Our World From Every Angle CJASN Clinical Journal of the American Society of Nephrology

ASN Journal Disclosure Form

As per ASN journal policy, I have disclosed any financial relationships or commitments I have held in the past 36 months as included below. I have listed my Current Employer below to indicate there is a relationship requiring disclosure. If no relationship exists, my Current Employer is not listed.

V. Tesar reports the following:
Employer: General University Hospital in Prague; Consultancy: AstraZeneca, Bayer, Boehringer-Ingelheim, Calliditas, CSL-Vifor, GSK, Eli Lilly, Novartis, Otsuka, Roche, Stada, Swixxbiopharma, Travere, Vera; Honoraria: For consultancy as follows: AstraZeneca, Bayer, Boehringer-Ingelheim, Calliditas, CSL-Vifor, Eli Lilly, GSK, Novartis, Travere, Vera; and Advisory or Leadership Role: member of the of the steering committee of clinical trials sponsored by Calliditas, Novartis, Otsuka, Travere, Vera.

I understand that the information above will be published within the journal article, if accepted, and that failure to comply and/or to accurately and completely report the potential financial conflicts of interest could lead to the following: 1) Prior to publication, article rejection, or 2) Post-publication, sanctions ranging from, but not limited to, issuing a correction, reporting the inaccurate information to the authors' institution, banning authors from submitting work to ASN journals for varying lengths of time, and/or retraction of the published work.

Name: Vladimir Tesar
Manuscript ID: CJASN-2025-001116R2
Manuscript Title: Kidney function outcomes with complete remission of proteinuria in the PROTECT study of sparsentan in IgA nephropathy
Date of Completion: October 15, 2025
Disclosure Updated Date: September 9, 2025
